# Local Insulin-Derived Amyloidosis Model Confronted with Silymarin: Histological Insights and Gene Expression of MMP, TNF-α, and IL-6

**DOI:** 10.3390/ijms23094952

**Published:** 2022-04-29

**Authors:** Katia Azarfar, Parichehreh Yaghmaei, Mahsa M. Amoli, Nasim Hayati-Roodbari, Azadeh Ebrahim-Habibi

**Affiliations:** 1 Department of Biology, Faculty of Basic Sciences, Science and Research Branch, Islamic Azad University, Tehran 1477893855, Iran; katiaazarfar@ymail.com (K.A.); hayati@srbiau.ac.ir (N.H.-R.); 2 Metabolic Disorders Research Center, Endocrinology and Metabolism Molecular-Cellular Sciences Institute, Tehran University of Medical Sciences, Tehran 1411413137, Iran; amolimm@tums.ac.ir; 3 Biosensor Research Center, Endocrinology and Metabolism Molecular-Cellular Sciences Institute, Tehran University of Medical Sciences, Tehran 1411713137, Iran; 4 Endocrinology and Metabolism Research Center, Endocrinology and Metabolism Clinical Sciences Institute, Tehran University of Medical Sciences, Tehran 1411713137, Iran

**Keywords:** regular insulin, amyloidosis, aromatic compound, MMP2, TNF-α, IL-6

## Abstract

Amyloidosis is a heterogeneous group of protein deposition diseases associated with the presence of amyloid fibrils in tissues. Analogs of insulin that are used for treating diabetic patients (including regular insulin) can form amyloid fibrils, both in vitro and in vivo as reported in patients. The main purpose of this study was the induction of localized insulin-generated amyloidosis and the observation of *silymarin* effects on this process. In order to obtain amyloid structures, regular insulin was incubated at 37 °C for 24 h. Congo red absorbance and transmission electron microscopy images validated the formation of amyloid fibrils. Those fibrils were then injected subcutaneously into rats once per day for 6, 12 or 18 consecutive days in the presence or absence of *silymarin*, and caused development of firm waxy masses. These masses were excised and stained with Hematoxylin and Eosin, Congo red and Thioflavin S. Histological examination showed adipose cells and connective tissue in which amyloid deposition was visible. Amyloids decreased in the presence of *silymarin*, and the same effect was observed when *silymarin* was added to normal insulin and injected subsequently. Furthermore, plasma concentrations of MMP2, TNF-α, and IL-6 inflammatory factors were measured, and their gene expression was locally assessed in the masses by immunohistochemistry. All three factors increased in the amyloidosis state, while *silymarin* had an attenuating effect on their plasma levels and gene expression. In conclusion, we believe that *silymarin* could be effective in counteracting insulin-generated local amyloidosis.

## 1. Introduction

Amyloidosis is characterized by abnormal extracellular amyloid deposition of a fibrillary protein substance in a tissue. The term refers to a collection of systemic diseases, in which misfolding of soluble precursor proteins may result in infiltrative depositions, leading to disruption of the structure and function of the normal organs [1]. Deposits are highly organized fibrils that adopt a cross-beta super-secondary structure: the so-called “amyloid”. Due to the variety of affected protein aggregates and their biochemical, physiological and clinical implications, the actual clinical manifestations and prognosis of amyloid-related diseases can be largely different [2]. Amyloid fibrils are generally composed of 2–8 protofilaments, each 2–7 nm in diameter and 3 nm wide, with a height of 2–7 nm. Protofilaments often twist around each other to form the typical 5–15 nm wide fibrils.

One of the roughly 25 disorders categorized as amyloid diseases is injection amyloidosis [3]. In contrast to systemic amyloidosis, the generated amyloid tumors are localized deposits, usually accompanied by mild clinical symptoms [4]. Hemodialysis, chronic inflammation and infections, tuberculosis, and osteomyelitis are the most regular types of diseases caused by amyloid tumors. Sometimes, the patient may have no clinical symptoms, although the amyloid deposits exist in soft tissue, bladder or the respiratory and gastrointestinal tracts [5]. In diabetic patients, injection amyloidosis can occur due to accumulation of the insulin that they use as treatment.

In vitro, when insulin is heated at high temperature, low pH and high ionic strength, a series of structural changes occur in the protein, which leads to the formation of fibrillary structures [6]. In vivo, insulin molecules have been found in fibrillary form at the site of frequent insulin injections. Insulin fibril that is formed in vivo displays the characteristics of amyloid aggregates, including the capacity to bind to Congo red dye, reacting with Thioflavin S, and a generally unbranched fibrillary morphology [7]. Histologically, chronic active inflammatory changes as well as foreign body reactions with multinucleated giant cells are seen within and around the amyloid depositions. Inflammatory cells surrounding subcutaneous amyloid are unable to digest insulin; consequently, accumulated insulin peptides in soft tissues undergo aggregation with reactive fibrotic processes and eventually take the form of amyloid fibrils [8]. Insulin-derived amyloidosis manifests itself by the presence of a hard subcutaneous mass at the injection site [9]. The amyloid mass has also been referred to as an “amyloidoma” and “insulin ball” [10].

Despite the importance of this condition, limited studies have been performed with the use of animal models for local amyloidosis.

According to Chinisaz et al. [11], when incubated at 57 °C for 24 h, regular insulin can form amyloid fibril. After 21 consecutive days of subcutaneous injection of human insulin amyloid fibrils, local amyloidosis in mice was generated, and within the tumor, lipohypertrophy, which includes the amyloid fibrils, was present. Kheirbakhsh et al. [12] have used turmeric for the reduction of local amyloidosis lumps and reported that after 12 consecutive days of subcutaneous injection of insulin amyloid fibers on the left abdominal region of rats, masses were formed, while turmeric attenuated the local amyloidosis bulk. They showed that the addition of turmeric to the fibrils previous to injection resulted in masses with a significantly reduced size, as well as a less ordered cellular structure. In another study, Nakamura et al. [10] demonstrated that repeated injections of a high amount of insulin at the same site causes formation of insulin-derived amyloidoma, which inhibits insulin absorption. They reported that this effect was caused by amyloid fibrils in the absence of a granulomatous reaction, and adhesion to native human insulin and various insulin analogs was observed. In the present study, the generation of local amyloidosis in rats has been studied in more detail (injection of insulin or fibril, duration of injection, testing animal’s serum…). The resulting model can be used as a general representation of local amyloidosis, in a similar manner to the in vitro fibril formation of proteins’ model, as an indicator for the behavior of pathogenic proteins.

The effect of *silymarin,* a natural compound containing flavone ligands, has also been tested on the model. In general, polyphenolic compounds have been reported to inhibit amyloid formation of a variety of proteins by their interactions with different stages of the protein structure, as it progresses toward fibril formation [13,14,15,16,17]. *Silymarin* has also been shown to have anti-amyloid properties on various proteins, including A-beta (amyloid beta), which forms amyloid structures in patients with Alzheimer’s disease [18]. This compound was chosen based on the possibility of a generic anti-amyloid effect of polyphenolic compounds [19] and the fact that it is a well-established medicine [20] and has been approved for use in humans.

## 2. Materials and Methods

### 2.1. Animals

Male Wistar rats weighing 250 ± 300 g were housed at six per cage (42 × 26 cm), at temperature of 22 ± 2 °C, 50% humidity, under a 12/12 h light/dark cycle, (lights on from 8:00 AM to 8:00 PM). Animals had free access to standard pellet food and water. Animals were acclimatized for one week to the laboratory conditions before the start of the experiment. All experiments were performed in accordance with the international guidelines set out in the Guide for the Care and Use of Laboratory Animals (Institute of Laboratory Animal Resources, 1996) and approved by the local Research and Ethics Committee.

### 2.2. Experimental Groups

Five groups of the randomly selected rats received different types and amounts of the injections at three time of 6, 12 and 18 consecutive days (Table 1). All groups received normal pellet food and water during the experimental period. No particular inclusion or exclusion criteria were applied. All animals were used in subsequent analyses. Researchers were aware of the animal distribution in the groups for the whole period of the research.

### 2.3. Amyloid Preparation

In order to induce amyloids in insulin, we used regular human insulin (EXIR Pharmaceutical Co., Tehran, Iran), which was dissolved in 50 Mm potassium phosphate buffer (pH 7.4) to a concentration of 0.5 mg/mL, and incubated at 37 °C for 24 h while being stirred by Teflon magnetic bars, in the absence and presence of 0.5 mM *silymarin*. Congo red test of amyloid formation was performed in order to track fibril formation over time [21]. Transmission electron microscopy captured images were used as complementary proof of amyloid formation (TEM; CEM 902A Zeiss microscope; Carl Zeiss, Jena, Germany).

### 2.4. Histological Processing

All tissue biopsies were fixed in formalin, embedded in paraffin blocks, and cut into sections of about 5–6 µm thick. Hematoxylin and Eosin (H&E), Congo red, as well as Thioflavin-S staining were applied to each tissue block. A light microscope (Carl Zeiss AG, Oberkochen, Germany) and optical microscope (Vanix T, Olympus, Japan) were used to observe the tissue section. Congo red and Thioflavin-S were obtained from Sigma-Aldrich (St. Louis, MO, USA).

### 2.5. Biochemistry Measurements and Immunohistochemistry

MMP2, TNF-α, and IL-6 were measured in our study subjects. After 18 days of continuous injection, blood samples were taken from the heart of the rats. Serums were then separated by centrifugation at 25,200× *g* (1500 rpm) for 10 min at 4 °C. Serological assays were performed to assess the levels of specific inflammatory cytokines (TNF-α, IL-6) and matrix metalloproteinase (MMP2). The concentrations of IL-6, TNF-α and MMP2 were evaluated by a sandwich enzyme-linked immunosorbent assay (ELISA); (Zell Bio kit, Lonsee, Germany). Immunohistochemistry was performed against *MMP2*, *TNF-α*, and *IL-6* (Santa Cruz Biotechnology, Santa Cruz, CA, USA).

### 2.6. Statistical Analysis

Data were statistically calculated by SPSS with the use of one-way analysis of variance (ANOVA), followed by post hoc Tukey test. The values were reported as mean ± standard error. Level of significance was considered at *p* < 0.05.

## 3. Results

### 3.1. Model Developments

**Figure 1:**
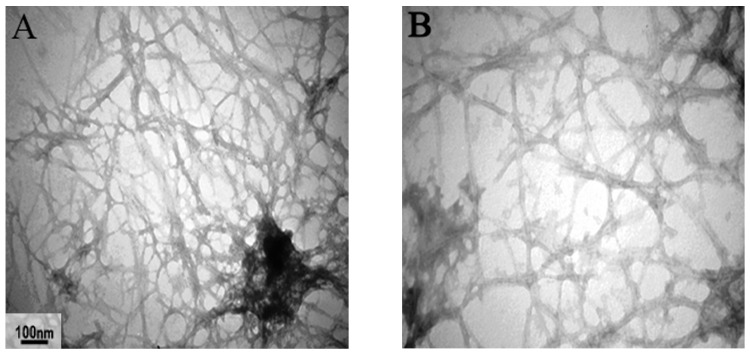

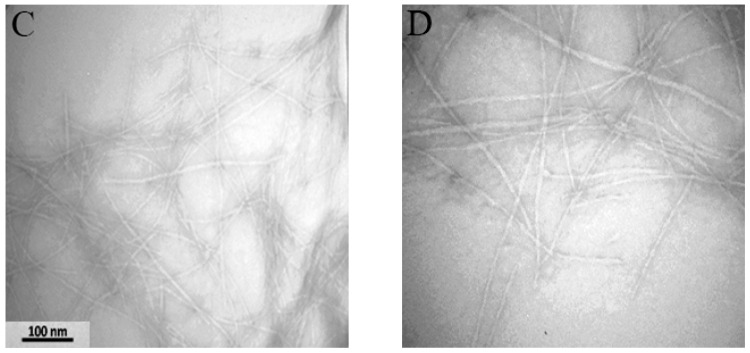
Transmission electron microscopy (TEM) image of regular insulin (1 mg/ml) incubated at pH 7.4 and 37 °C. The TEM image shows that amyloid fibril formed from regular insulin (**A**,**B**) and the effect of (0.5 mM) silymarin (**C**,**D**) on insulin amyloid aggregation.

The rate of insulin amyloid fibril formation in vitro could be affected by several factors such as pH, temperature, protein concentration, and the presence of denaturants [22]. In our setting, incubation at 37 °C at physiological pH and stirring at 100 rpm led to insulin fibrils formation after 24 h. Fibril formation was followed over time by taking samples at 0–24 h of incubation and observing the absorbance spectrum in the presence of Congo red (Supplementary Figure S1a). Redshifts, which are indicative of amyloid structure presence, were observed sooner for insulin alone (i.e., after 12 h of incubation), while in the presence of silymarin, this shift was observed after 24 h. Fibril formation was also checked by TEM after 24 h and was found to be less when silymarin was present (Figure 1A,B). An additional experiment was performed to check the possibility of silymarin binding to fibrils by reading absorbance at 320 nm which is related to silymarin presence (Supplementary Figure S1b). For supernatants of formed fibrils to which silymarin was added, no absorbance was detected at this wavelength, thus indicating the possibility of an affinity between the compound and fibrils.

In the next stage, fibrils were injected into rats for three different durations. After 6, 12, or 18 days, the control group, which had been injected with potassium phosphate buffer, did not show any apparent change around the injection site. In the sham groups, which had a daily dose of insulin (Sham1), and 500 µL insulin fibrils (Sham2), abnormal mass formation was observed around the injection site. These masses were waxy adipose bodies, white-yellow in color (Supplementary Figure S2), and similar to the lipohypertrophic bodies observed in human diabetic patients; these are solid firm tumors and are usually diagnosed by palpation [23]. Patches of abnormal lumps formed in Sham2 were bigger and larger than in Sham1. These lumps were even extended to the back of the animal, which there became lighter in color and smaller than the lumps formed at the injection site (results not shown). This is indicative of a more pronounced effect of amyloids compared to normal protein. Formation of insulin amyloid masses in all groups that had 6-days injection of either insulin or fibril of insulin was remarkably less than 12- and 18-day groups, as assessed by the formed mass volume and diameter (Supplementary Figure S2). Animals receiving 18-day injection showed the largest masses. In all sham groups, the effect of receiving amyloid fibrils was worse than normal insulin.

In the Exp1 group, the presence of *silymarin* resulted in an observable reduction in amyloid accumulation compared to Sham1, furthermore, in this group, the amyloid masses color was pale yellow. Similarly, in the Exp2 group, which had received insulin amyloid formed in the presence of *silymarin*, a decrease in the amount of amyloid masses was observed. In vitro, *silymarin* was also shown to attenuate insulin fibrils (Figure 1C,D).

If insulin or insulin fibril was accompanied by *silymarin* when injected into rats, the amount of formed lumps was greatly reduced, an effect that was more pronounced in the Exp1 group.

Overall, insulin fibril may cause necrosis in the surrounding site of insulin injection, and the presence of the polyphenol containing *silymarin* reduces insulin fibril formation and results in a smaller amyloid mass in vivo.

### 3.2. Tissue Staining and Analysis

**Figure 2:**
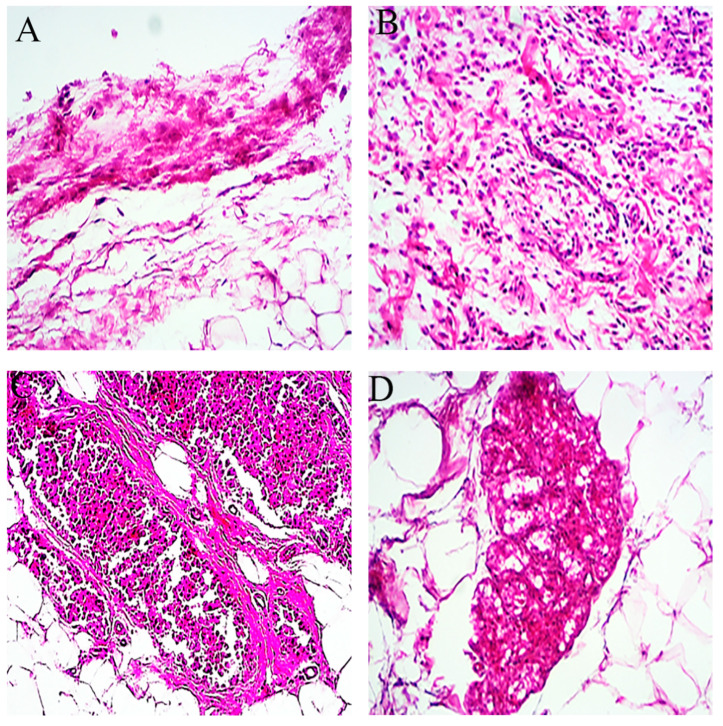

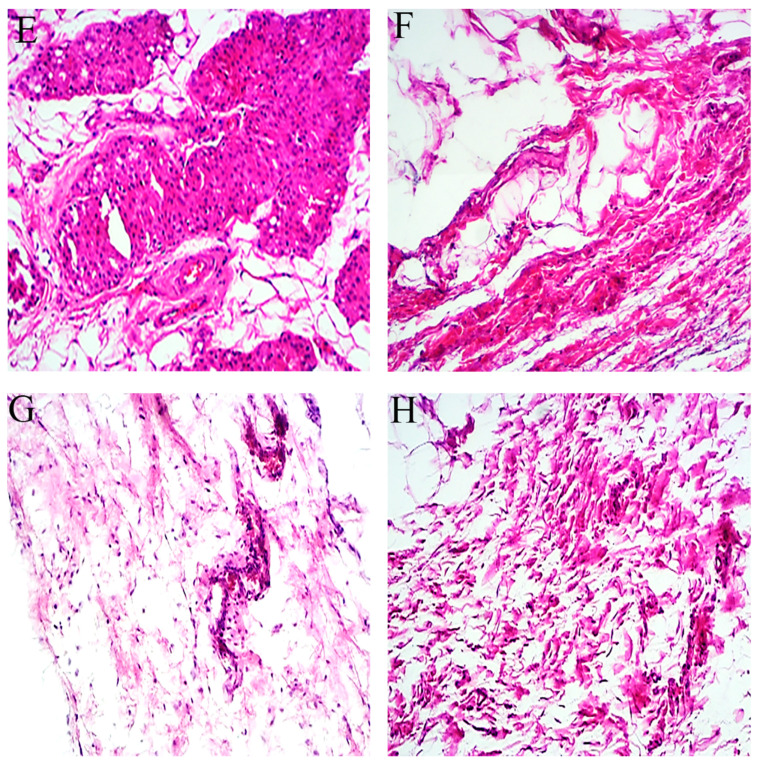

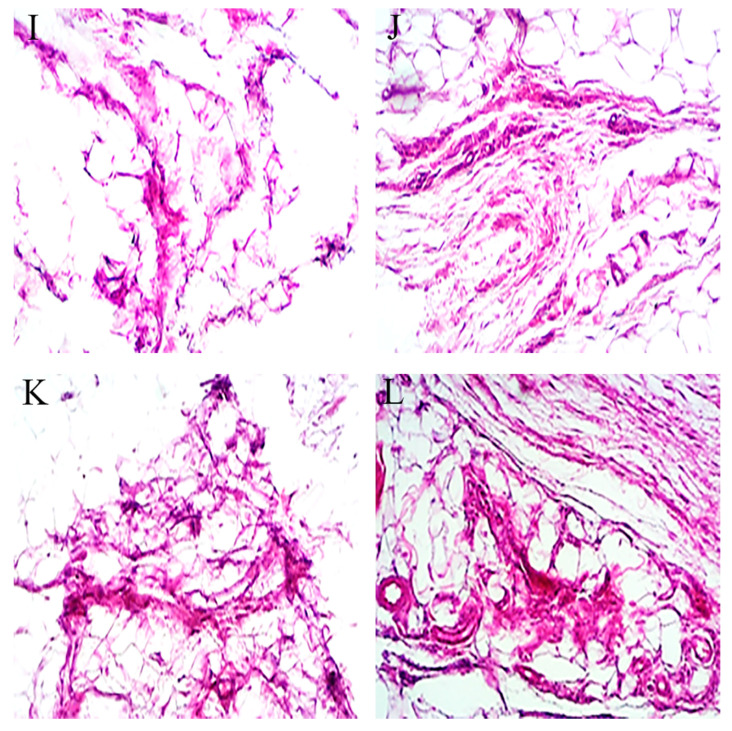
Light microscopic results of excised mass, revealing amyloid fibril deposits by Hematoxylin and Eosin (H&E) staining. (**A**–**L**) are ×40 magnifications, where Sham1 (**A**–**C**), Sham2 (**D**–**F**), Exp1 (**G**–**I**), and Exp2 (**J**–**L**) are represented. In each group, results of 6, 12, and 18 days of injection are shown from left to right (e.g., A: 6 days, B: 12 days, C: 18 days).

In order to further identify the characteristics of localized amyloid masses, generic and amyloid-specific dyes were used. With Hematoxylin and Eosin as a generic dye, amyloid aggregates appeared as amorphous, eosinophilic extracellular material surrounded by connective tissue (Figure 2). Amyloid fibrils are seen here as light purple deposits that have been analyzed quantitatively to some extent (Supplementary Figure S3). However, the deposits cannot be precisely differentiated from fibrosis, muscle tissue, or hyalinosis, specific dyes were used in the next step.

**Figure 3:**
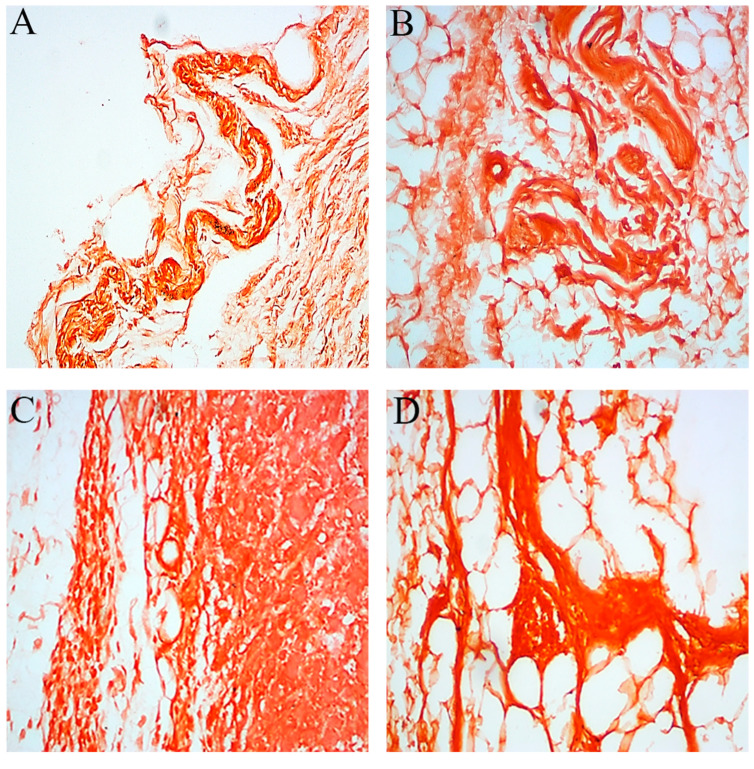

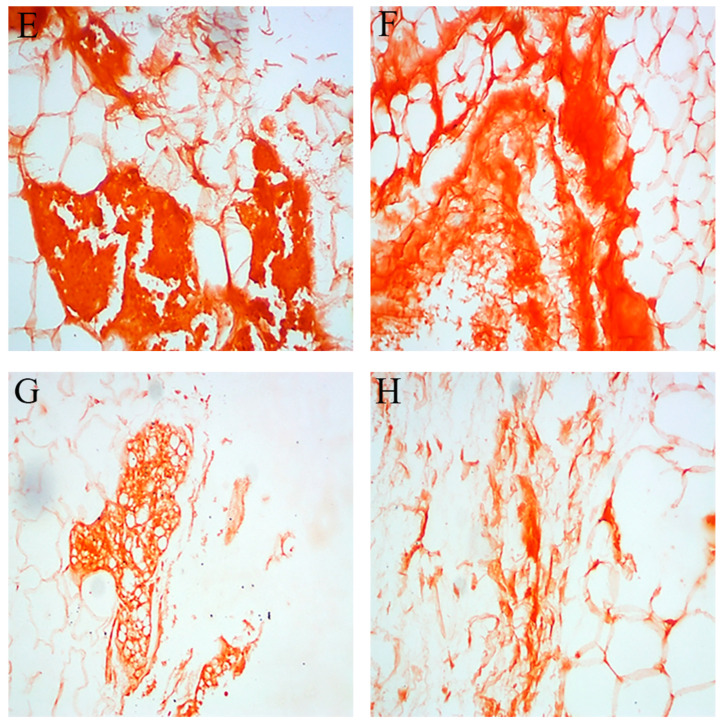

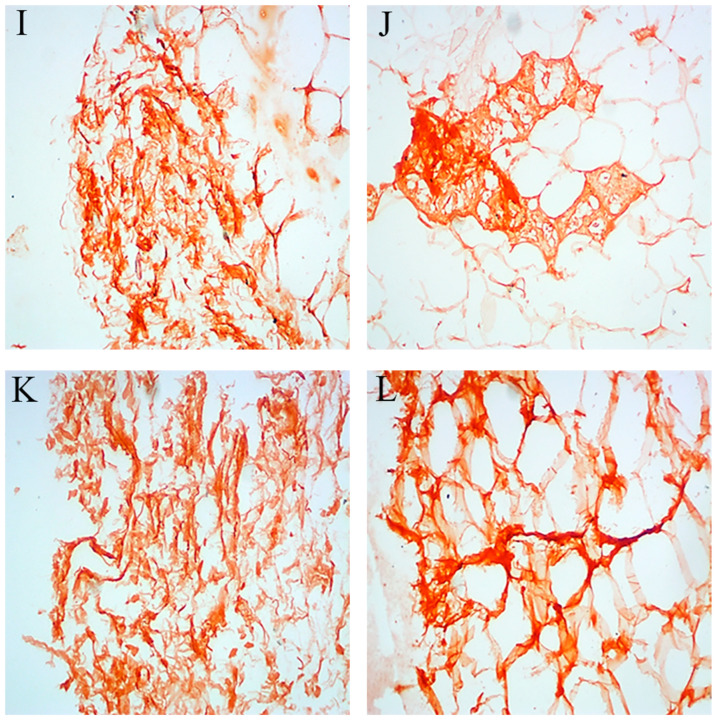
Light microscopic results of excised mass, revealing amyloid fibril deposits by Congo red staining. (**A**–**L**) are ×40 magnifications, where Sham1 (**A**–**C**), Sham2 (**D**–**F**), Exp1 (**G**–**I**), and Exp2 (**J**–**L**) are represented. In each group, results of 6, 12, and 18 days of injection are shown from left to right (e.g., A: 6 days, B: 12 days, C: 18 days).

Congo red is the most widely used stain for amyloid fibrils characterization, in which amyloids manifest themselves as pink or red stains under an ordinary light microscope [21]. It is an organic compound able to crosslink with cross-beta structures of amyloid fibrils [24]. Here, the amyloid fibrils are seen as dark-shade salmon pink deposits (Figure 3), and an increase in amyloid fibrils could be seen with increasing injection days. Congo red can also stain hyaline deposits, elastin, and collagen [25], and in order to better characterize the aggregates, an additional probe, namely Thioflavin-S, was also used.

**Figure 4:**
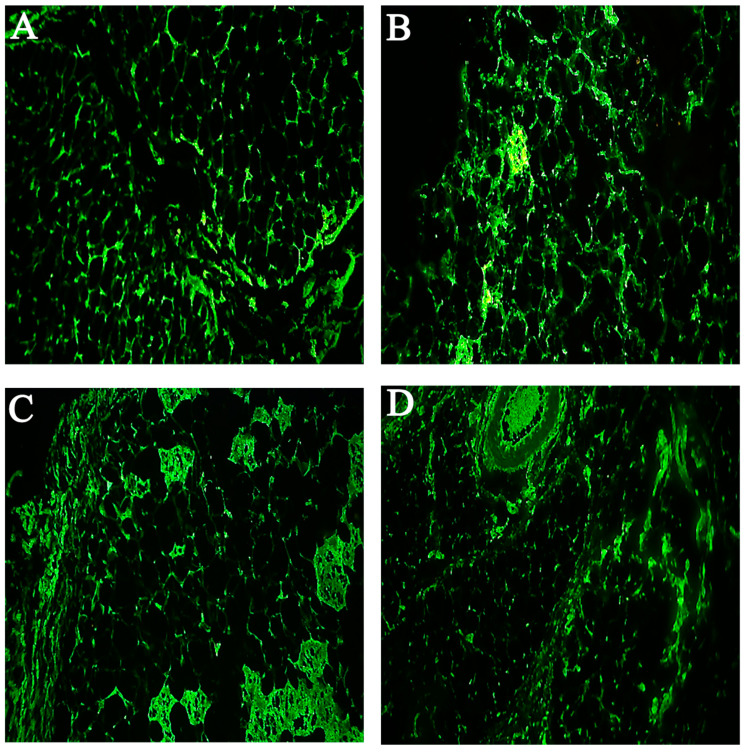

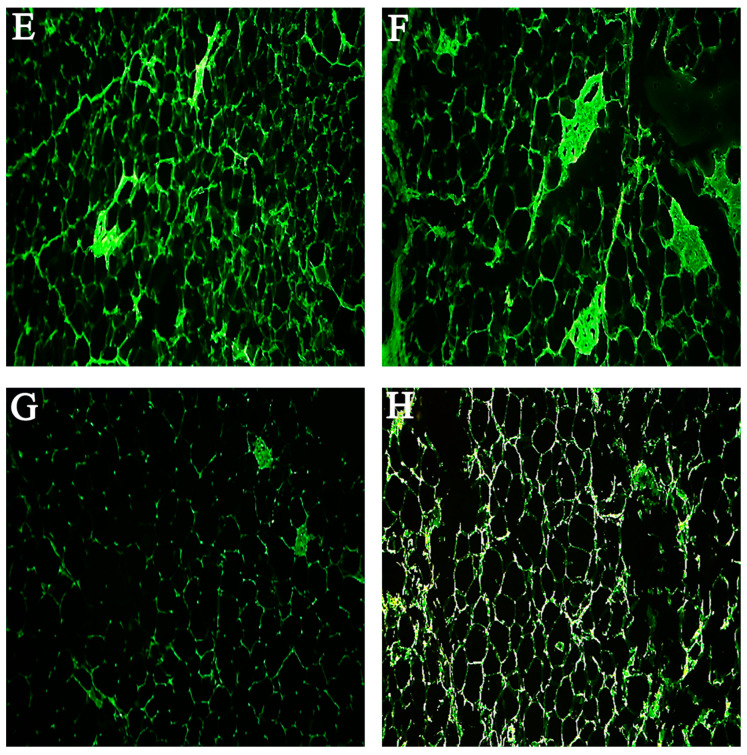

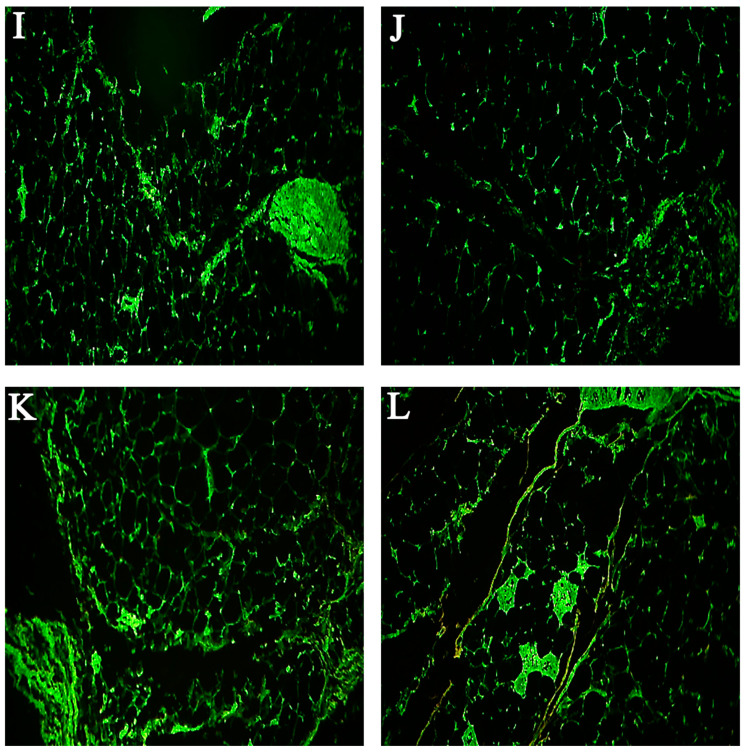
Fluorescence microscopy results of excised mass, revealing amyloid fibril deposits by Thioflavin-S staining. (**A**–**L**) are ×40 magnifications, where Sham1 (**A**–**C**), Sham2 (**D**–**F**), Exp1 (**G**–**I**), and Exp2 (**J**–**L**) are represented. In each group, results of 6, 12, and 18 days of injection are shown from left to right (e.g., A: 6 days, B: 12 days, C: 18 days).

Thioflavin is a small molecule with a high affinity for beta structures. It is useful in identifying amyloid fibers and produces strong fluorescence after binding to amyloid fibers [26,27]. This probe has been used in other studies with a similar setting (subcutaneous insulin injection in animals) for detecting the amyloid structures, sometimes alongside with Congo red [28]. In some human case studies, Congo red alone has been used to characterize insulin-derived amyloids (e.g., [23]), although it is preferable to use several probes to check on amyloids [21]. As shown in Figure 4, upon adding Thioflavin-S, amyloid deposits appear as bright yellow-green particles under a fluorescence microscope. Here as well, increasing injection days caused an increment in amyloid mass density and the formation of new vessels (Supplementary Figure S4).

### 3.3. Analysis of Metalloproteinase MMP2, TNF-α, and IL-6 Cytokines Concentration in Serum and Expression in Amyloid Masses of 18-Day Injected Rats

Inflammation is a response of living tissue to local injury. According to Azevedo [29], the accumulation of amyloid fibrils causes tissue damage and elicits pro-inflammatory cytokine production and nonlocal immune cell infiltration into tissues. Cytokine production is an important pathological event in the development of inflammatory cascades.

**Figure 5:**
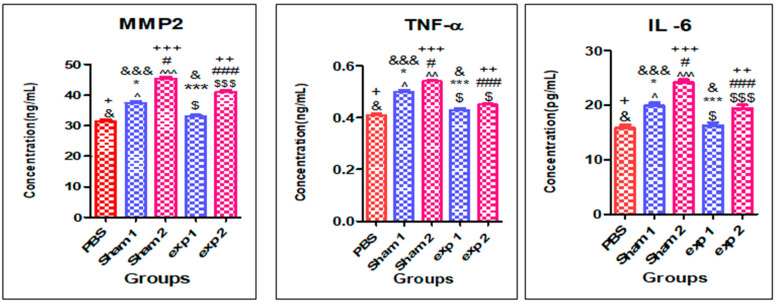
MMP2, IL-6 and TNF-α levels in plasma as measured by ELISA. (* *p* < 0.05), (*** *p* < 0.001) show the difference from Sham1; (# *p* < 0.05), (### *p*< 0.001) show the difference from Sham2; (& *p* < 0.05), (&&& *p* < 0.001) show the difference from PBS group; (+ *p* < 0.05), (++ *p* < 0.01), (+++ *p* < 0.001) show the difference from PBS group; (^ *p* < 0.05), (^^ *p* < 0.01) and (^^^ *p* < 0.001) show the difference from Sham1 (MMP2, IL-6, TNF-α); ($ *p* < 0.05) and ($$$ *p* < 0.001) show the difference from Exp1) MMP2, IL-6, TNF-α). (PBS is the control group and Exp1 and Exp2 are experimental groups #1 and 2 respectively).

Our histological experiments have shown that formation of amyloid deposits due to consecutive insulin injection resulted in tissue abnormalities. This accumulation would also trigger an immune response and possible chronic inflammation. This assumption was tested by measuring inflammatory cytokines (TNF-α, IL-6) and matrix metalloproteinase (MMP2), in the 18-day injected rats in which the largest masses had been observed. Serum level measurements showed a significant increase in all these factors in both the Sham1 and Sham2 group (Figure 5). In all cases, the Sham2 group showed elevated levels in comparison with Sham1, which could be indicative that the fibrillary form of insulin causes more damage and inflammation. Overall, these results show that formation of large amyloid masses at the injection site under the skin can lead to acute inflammation and that amyloid injections elicit a higher response compared with native insulin.

**Figure 6:**
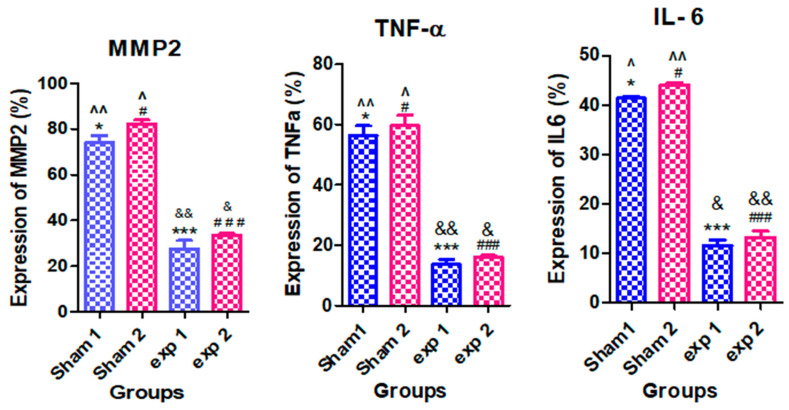
Comparison of expression of MMP2, TNF-α, and IL-6 proteins was induced in adipose tissue cells at the subcutaneous injection site. Results are presented as mean ± SE for 4 rats (n = 4). (* *p*<0.05), (*** *p* < 0.001) show the difference from Sham1; (# *p* < 0.05), (### *p* < 0.001) show the difference from Sham2; (^ *p* < 0.05) and (^^ *p* < 0.01) show the difference from Sham2; (& *p* < 0.05) and (&& *p* < 0.01) show the difference from Exp2. (Exp1 and Exp2 are experimental groups #1 and 2 respectively).

Conversely, the Exp1 group shows no increase in serum levels of MMP2, IL-6 and TNF-α in comparison with the control group, but compared to the sham group, a significant difference was observed, which could be related to the effect of *silymarin*, indicating that the use of *silymarin* with native insulin can prevent injection-related inflammation. Comparison of the Exp2 group with the control is indicative of an increase in inflammation-related parameters, and there is a difference with Exp1 group, but the levels are still significantly less than the Sham2 group, which is overall indicative of attenuation of inflammation. The next experiment was to evaluate the expression of *MMP2*, *TNF-α*, and *IL-6* genes in the masses in order to check whether the presence of cytokines could be detected locally. In line with the results obtained in serum measurements, in the sham groups, all those gene products could be detected at the tissue level, while the presence of *silymarin* could decrease them substantially, as shown in the Exp1 and Exp2 group results (Supplementary Figures S5–S7 and Figure 6). Comparison of Sham1 and Sham2 shows that the fibrillary form of insulin has a more pronounced effect on local gene expression of inflammatory cytokines. Similarly, the Exp2 group still shows higher levels of those cytokines when compared to the Exp1 group.

## 4. Discussion

Local cutaneous amyloidosis is formed by extracellular accumulation of amyloid deposits in the dermis. Apoptosis of keratinocytes coupled with fibril degradation is the cause of this amyloid formation, and eosinophilic, amorphous, and fissured substances are found in the dermis and subcutaneous tissue [30,31]. Localized cutaneous amyloidosis usually presents as a tumor-like lesion, but several nodules may develop.

Continuous subcutaneous injection of insulin may become associated with cutaneous amyloidosis [10]. Recognized as “Alns”(amyloid in the insulin injection site) by the nomenclature committee of the International Society of Amyloidosis [32], this type of localized iatrogenic insulin-induced amyloid was first recorded in 1983 [33], while it was identified in a diabetic patient after five weeks of continuously using porcine insulin. Since then, 75 cases have been reported in patients using a wide range of insulin dosage forms, and their incidence rate is constantly increasing.

Many diabetic patients with a long history of the disease have a solid mass in the abdomen, which could be detected during physical examination. Masses have also been reported in other areas such as the thighs, arms, chest, and lymph nodes [34,35].

Considering the high prevalence of diabetes mellitus and insulin treatments, the incidence rate of cutaneous amyloidosis might have been underestimated. This is also a problematic issue in diabetes control, since Nagase et al. [9] have shown that only 34% of injected insulin is absorbed from a site with amyloid deposits. Furthermore, insulin amyloid deposition reaction with anti-insulin antibodies results in a positive staining result [34]. Adhesion of insulin amyloid to native insulin and amyloid formation or degradation of newly injected insulin could be the causes of impaired insulin uptake after the formation of amyloid masses [10].

At the molecular level, it has been shown that the dissociation of insulin hexamers and dimers into the monomeric form is a key step toward the formation of insulin fibrils and that both A and B chains of insulin are amyloidogenic [36,37]. Insulin fibrils could be formed in a variety of conditions in vitro, but their injection in vivo is needed to mimic the real condition to some extent.

Our results showed that injecting insulin or fibrillar insulin for 6 consecutive days resulted in a smaller mass formation compared with the group treated for 12 and 18 days. We also found that the color of the masses, which were initially light yellow, turned into dark yellow with increased injection days, and that an expansion of the masses could also be seen: while there was as injection into the abdomen, masses could also be detected on the back of the animal. This observation is worth further investigation since it happened over the longer period of injection. Thus far, spreading has been observed in the case of amyloid-beta oligomers, which have aggregated in other parts besides their injection site into the brain [38].

In previous works, injection duration varied between one to three weeks. Nakamura et al. [10] could achieve rapid amyloid formation in C57BL/6 mice with seven-day injections of insulin amyloids at high concentration. Chinisaz et al. [11] and Kheirbakhsh et al., [12] conversely, reported the formation of waxy body amyloid masses as the result of daily subcutaneous injection of amyloid fibers to NMRI mice and male Wistar rats, respectively, for 21 consecutive days. In a recent study, Metkar et al. [28] used male Wistar albino rats and a period of 14-day injections. Breed and concentration of the injected substance could be of importance in the mass formation process. Previously, the counteracting effect of turmeric and two serine proteases have been shown toward subcutaneous insulin amyloid formation [12,28].

Localized amyloidosis can affect any part of the body. Hemodialysis, chronic inflammation and infections, tuberculosis, and osteomyelitis are the most regular types of diseases caused by amyloid tumors [30]. Sometimes, the patient may have no clinical symptoms, although the amyloid deposits exist in soft tissue, bladder or the respiratory and gastrointestinal tracts [5]. Baumgart et al. [39] have stated that immunoglobulin-induced (AL) light chain amyloidosis of the lungs and bronchi can occur both systematically and locally. Besides the lungs and bronchi, amyloids can be found in the bladder, kidneys, and heart [40]. As Fuah et al. [41] have stated, in localized amyloidosis, it is not uncommon to find a system that is more prominently affected than others. Common sites of involvement include the kidneys, liver, heart, peripheral nerves, musculoskeletal system, and skin.

Concerning *silymarin*, Yaghmaei et al. [42] have reported the anti-amyloid effect of the compound on Aβ plaques in vivo. Guo et al. [18] have mentioned that *silymarin* is able to control Aβ production by inhibiting the gene expression of its precursor (APP), while also preventing the polymerization of Aβ. Mahdavimehr et al. [43] have shown that *silymarin* effectively reduces the formation of amyloid fibrils of lysozymes (in vitro) in a concentration-dependent manner. Protein samples incubated in the presence of *silymarin* were free of amyloid fibril, and only small amounts of amorphous protein were observed.

A common feature linked to amyloid disease is inflammation. Reports have shown that chronic inflammatory diseases such as rheumatoid arthritis may led to amyloidosis, and this is related to an increase in serum amyloid A protein in the liver [44,45]. Conversely, Azevedo et al. [29] have shown that accumulation of amyloid fibrils leads to tissue damage and results in the production of proinflammatory cytokines. This condition causes a vicious cycle, which can increase amyloid production and create an environment for chronic inflammation. Ultimately, affected tissues will deteriorate and lose their normal function. According to Alasmari et al. [46], there is a clear relationship between the production of neuronal cytokines and the progression of Alzheimer’s disease.

Thus far, we are not aware of other studies investigating inflammation-related parameters in subcutaneous insulin-derived amyloidosis. Our study has shown that the levels of MMP2, IL-6 and TNF-α increase in this condition, both at serum level and in the injection site.

The activity and expression of the MMP gene is increased by the secretion of pro-inflammatory cytokines, caused by various collagens and gelatins breakdown, which is exacerbated by inflammation. MMP2 levels are also increasing in inflamed tissues as well as in the case of neuropathic pain. Several mediators have roles here, such as interleukin-1 beta, TNF-α, and nitric oxide [47].

When *silymarin* was added to insulin, we observed an attenuating effect of these parameters, and even a preventive effect in the case of non-fibrillary insulin. *Silymarin* can act via its anti-inflammatory effect, which has been reported in numerous studies in animal models of diseases, including rheumatoid arthritis in a collagen-induced arthritis rat model [48], renal carcinogenesis [49], colon ulcer [50], and metabolic syndrome [51], as examples. In humans, the results of experimental research have shown an anti-inflammatory effect for *silymarin* when used in cases of liver pathologies such as non-alcoholic fatty liver disease [52] or metabolic-associated fatty liver disease [20]. The anti-inflammation effect of *silymarin* administration could be related to an inhibition of the nuclear factor kappa B (NF-κβ) pathway. Besides the fact that NF-κB is activated in different types of liver pathologies [53], and *silymarin* is found to affect pro-inflammatory signals that are derived from NF-κB (e.g., TNF-α, and IL-6) [54], the results of a number of in vitro studies also point out this pathway. For example, in mesangial human cells, *silymarin* has specifically inhibited TNF-α-induced calcium-dependent NF-κB [55], and in a pancreatic beta cell line, it has inhibited cytokine-stimulated activation of NF-κB [56]. Overall, the effect of *silymarin* on NF-κB seems to be specific, and in many instances, a dose and time dependency has also been observed, as demonstrated through numerous examples in an extensive review [57].

Further than its anti-inflammatory effect, *silymarin* is also able to lessen in vitro fibril formation of various proteins such as Abeta [58], lysozymes [43] and insulin (as shown here), and its effect on the inflammatory state of the animals could also be related to a general attenuation of amyloid formation. There remains a question of how *silymarin* may affect insulin’s therapeutic effects in vivo. The compound is mainly used as a medicine in liver pathologies, but it has been suggested to be useful in diabetic states. According to a recent extensive review, it appears to reduce insulin resistance [59], while other reviews highlight its potential to counteract metabolic syndrome or diabetes complications (including, for example, an inflammatory state) [60,61,62].

## 5. Conclusions

In conclusion, after the induction of insulin-related localized amyloidosis, we found a local increase in inflammatory related proteins, as well as a general increase in these parameters in the serum of affected animals. Concomitant use of *silymarin* with insulin can attenuate the formation of amyloid masses, and the presence of this compound decreases insulin amyloid structure formation. We suggest that this and similar studies may develop a proposal on various compounds that could be used to counteract the underestimated problem of insulin-generated amyloidosis.

## Figures and Tables

**Table 1:** Experimental design: description of the animal groups used in the study.

1. Control group	Received (500 µL) daily subcutaneous injections of potassium phosphate buffer (insulin amyloid vehicle)
2. Sham1 (control group)	Received (500 µL) daily subcutaneous injections of insulin
3. Sham2 (control group)	Received (500 µL) daily subcutaneous injections of amyloid fibrils
4. Exp1 (experimental group1)	Received (500 µL) daily subcutaneous injections of insulin and 0.5 mM *silymarin* (70 mg/kg/day) in potassium phosphate buffer (pH 7.4)
5. Exp2 (experimental group2)	Received (500 µL) daily subcutaneous injections of amyloid fibrils formed in the presence of 0.5 mM *silymarin* (70 mg/kg/day) in potassium phosphate buffer (pH 7.4)

## Data Availability

All data are available within the manuscript and supplementary materials. The raw data are available on request from the corresponding author.

## Supplementary Materials

The following supporting information can be downloaded at: https://www.mdpi.com/article/10.3390/ijms23094952/s1.
