# Supplementary material for: Local Insulin-Derived Amyloidosis Model Confronted with Silymarin: Histological Insights and Gene Expression of MMP, TNF-α, and IL-6
Source: Int J Mol Sci. 2022 Apr 29;23(9):4952. doi: 10.3390/ijms23094952 (PMC9101448; doi:10.3390/ijms23094952)
Supplement: Supplementary file 1 [file ijms-23-04952-s001.zip › ijms-1650361-supplementary.pdf]

Supplementary Figures:

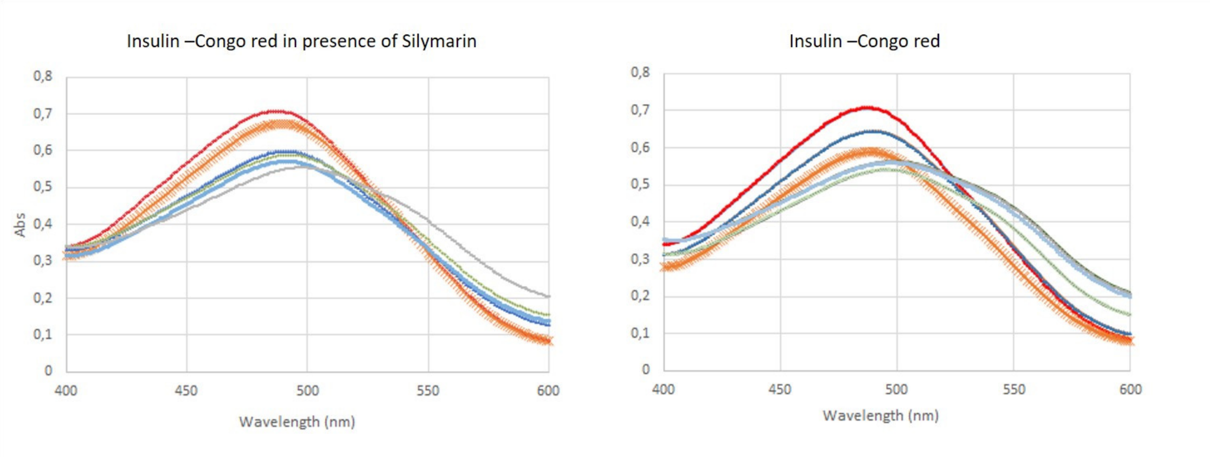

Supplementary Figure S1a. Congo red test of amyloid formation over time. Absorbance spectrum of insulin solutions incubated over time in presence (left panel) or absence (right panel)of silymarin, when Congo red is added to the solution. Redshift is indicating of amyloid structures presence (fibril formation)

● Congo red alone, × : at time 0, ▲ : after 4hours, ◆ : after 12 hours, ■ : after 20 hours, -- (light grey): after 24 hours.

Concentration microgram/ml	Abs 320,0 nm
4	0,026
8	0,069
10	0,071
12	0,091
16	0,118
20	0,124
24	0,115
32	0,162
40	0,28
50	0,286
60	0,414
80	0,488
100	0,542

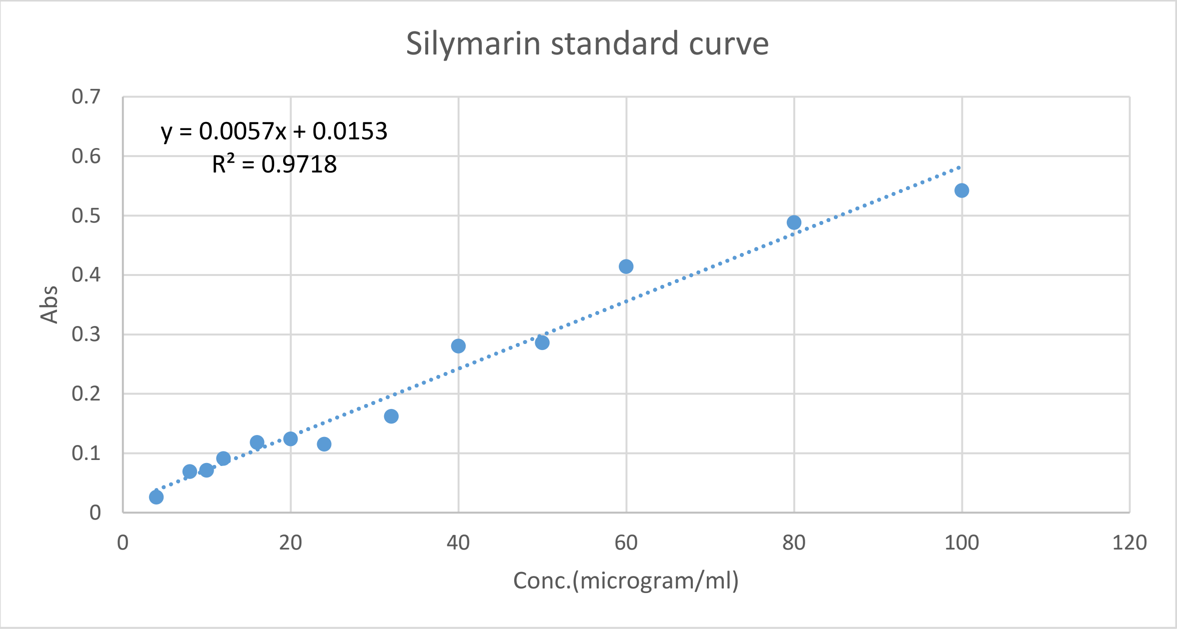

Supplementary Figure S1b. Results of measuring Silymarin's different concentrations (4–100 microgram/ml) absorbance at 320 nm.

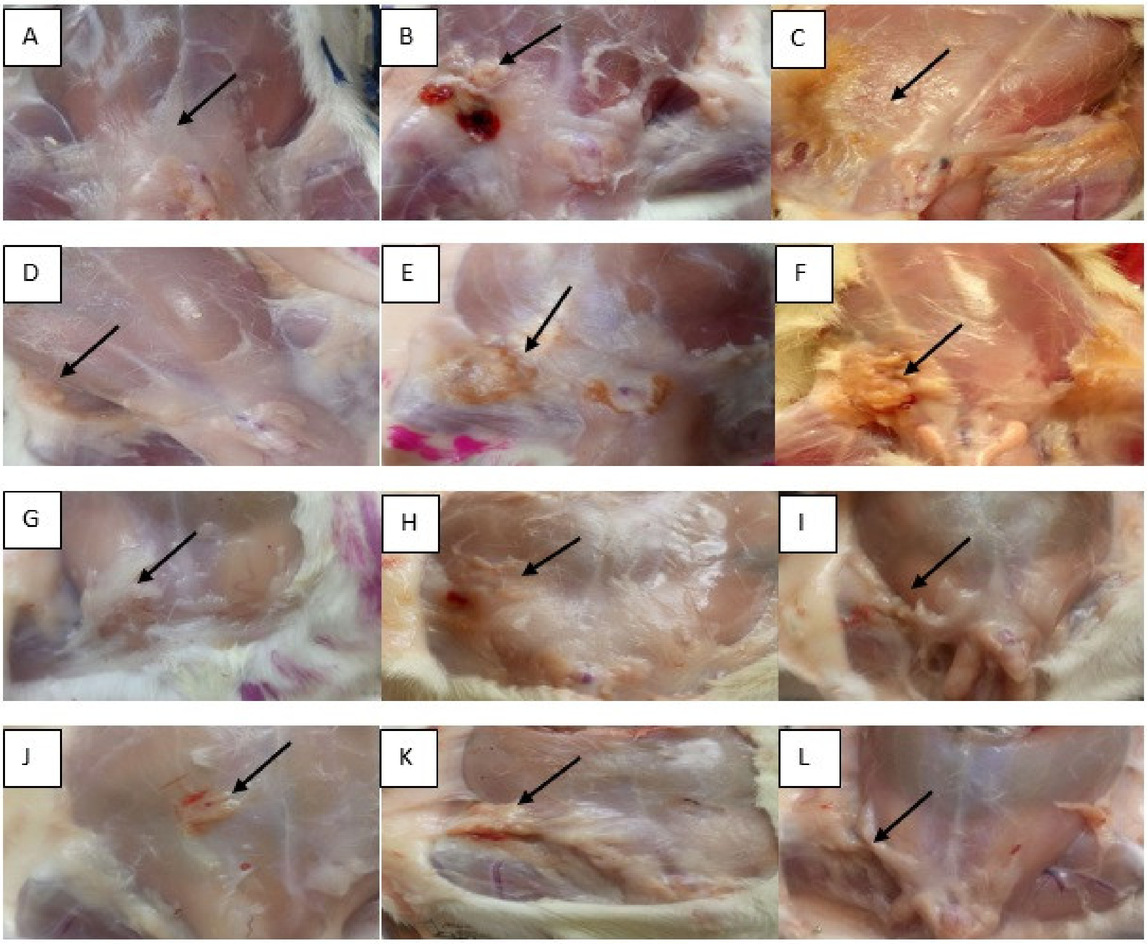

Supplementary Figure S2. Morphological studies related to injection of regular insulin in the subcutaneous area of the abdomen. Sham1(A, B, C), sham2(D, E, F), exp1(G, H, I), exp2(J, K, L). All groups show (6), (12), (18) days of injection from left to right . Arrows show amyloid deposits.

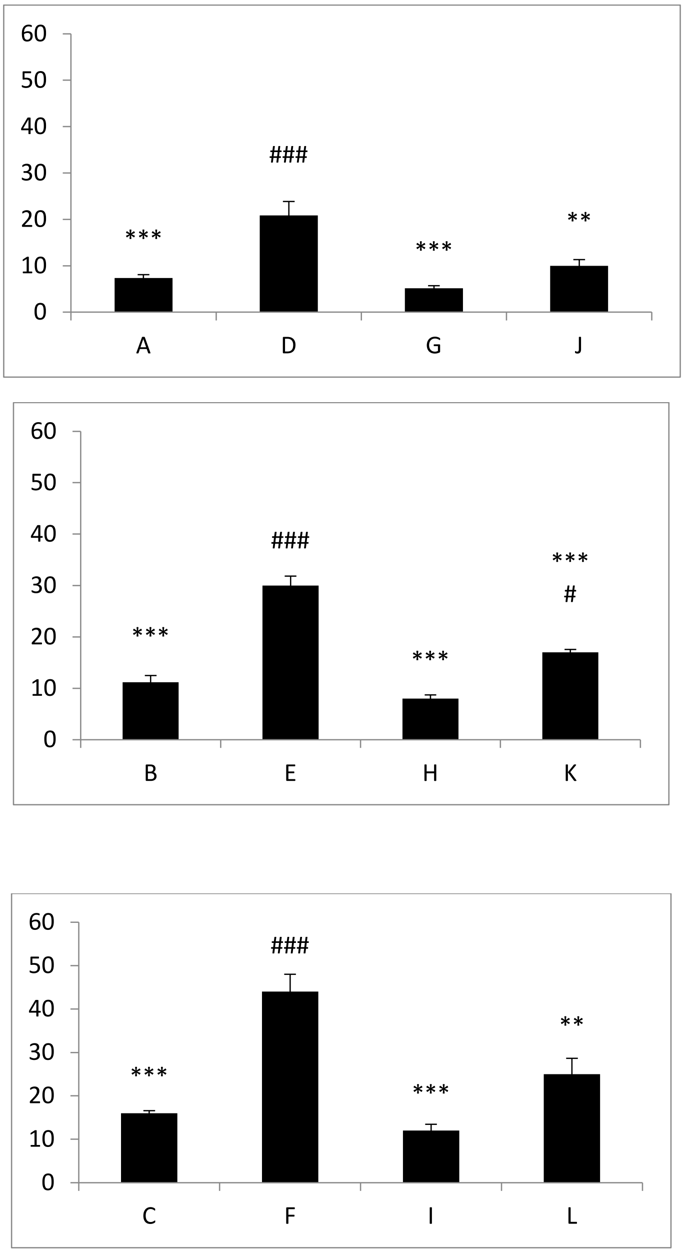

Supplementary Figure S3. Analysis of the H&E staining (Fig.3 of the main manuscript) by Image J. Numbers are indicative of areas that correspond to amyloid deposits in the original image. A-J: 6-days injection of insulin (A), insulin fibrils (D), insulin+ Sylimarin (G), and insulin fibrils+Sylimarin (J). B-K: 12-days injection of insulin (B), insulin fibrils (E), insulin+ Sylimarin (H), and insulin fibrils+Sylimarin (K). C-L: 18-days injection of insulin (C), insulin fibrils (F), insulin+ Sylimarin (I), and insulin fibrils+Sylimarin (L).

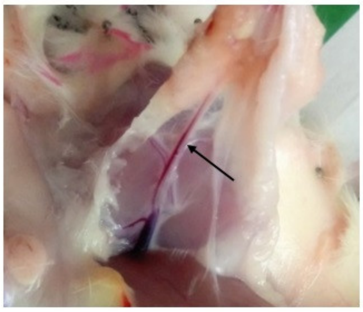

Supplementary Figure S4. Angiogenesis due to injection of amyloids.

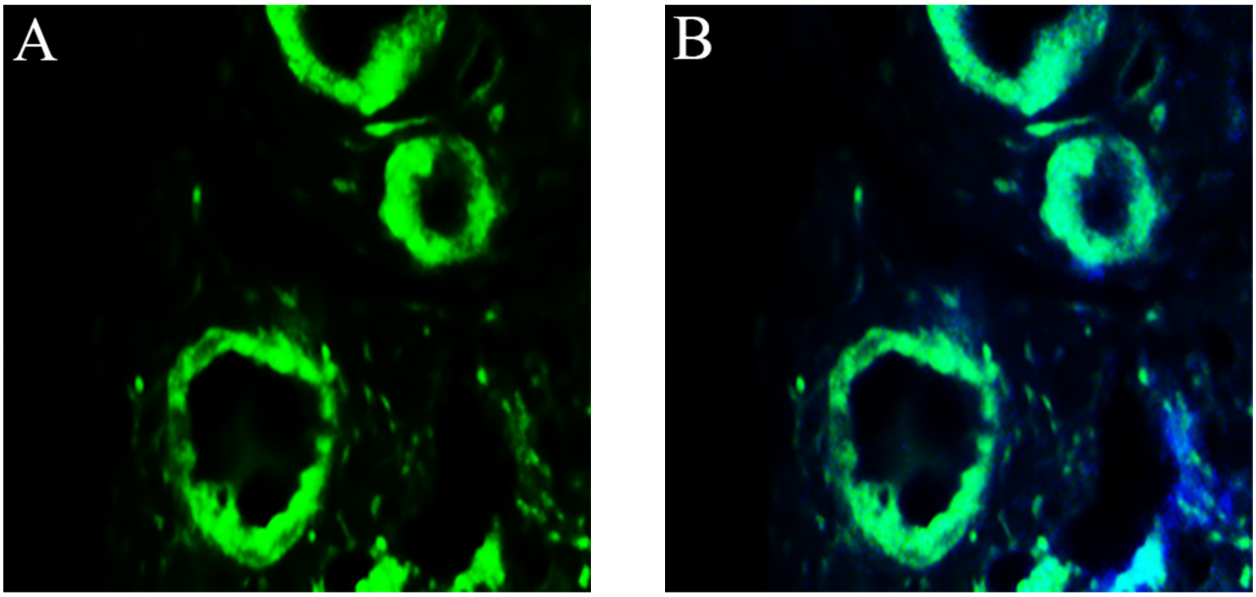

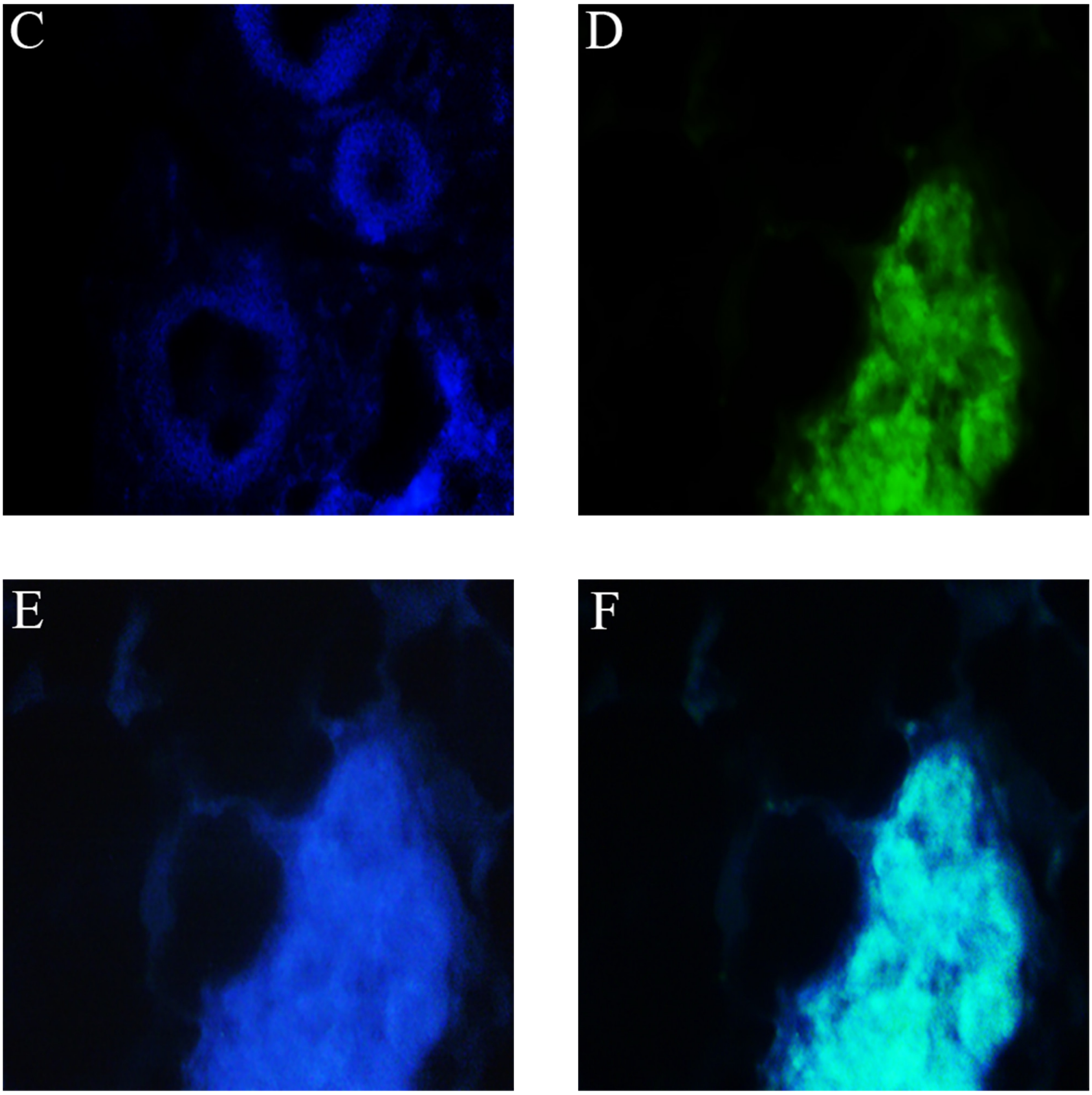
C
D
E
F

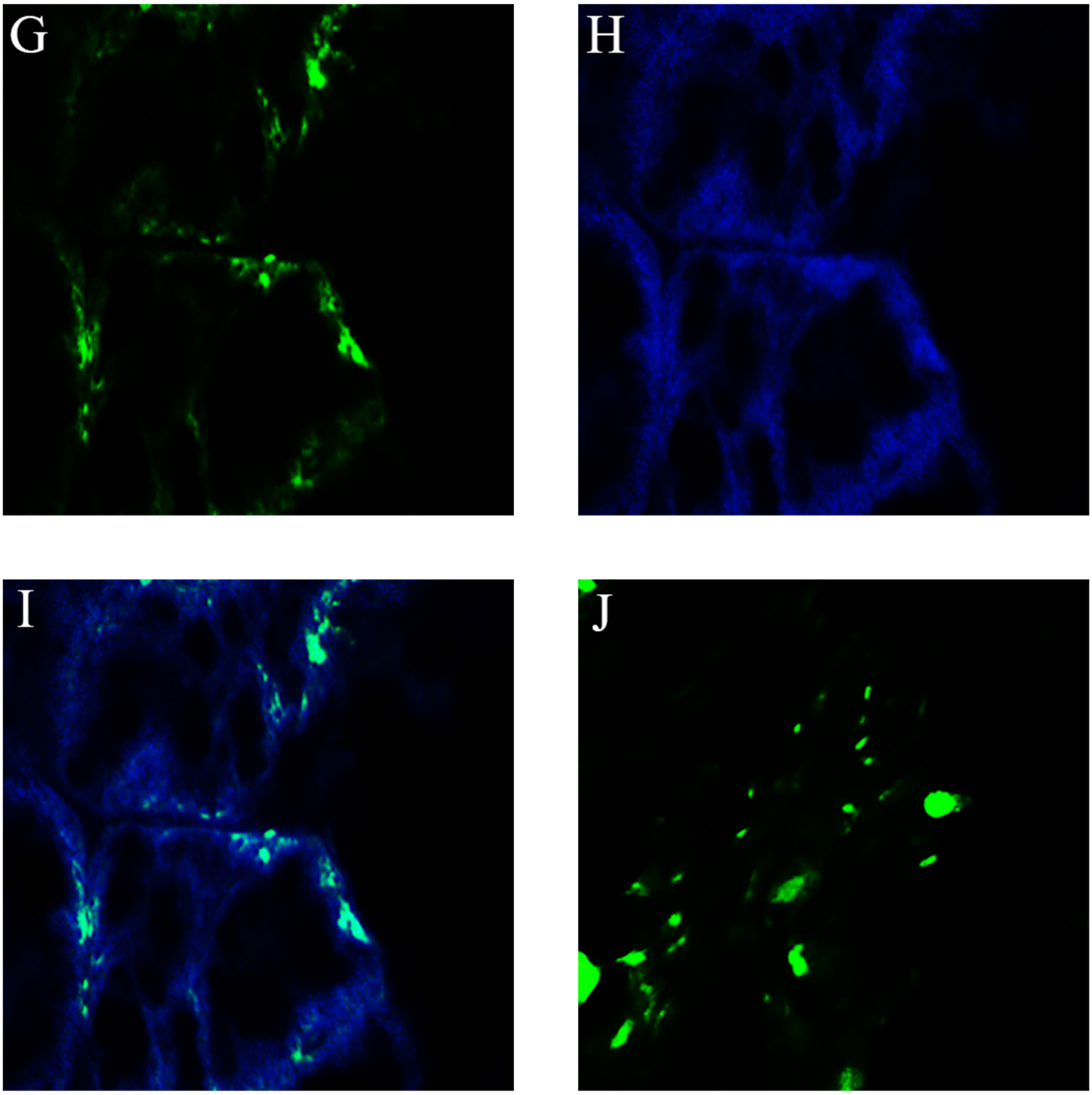
G
H
I
J

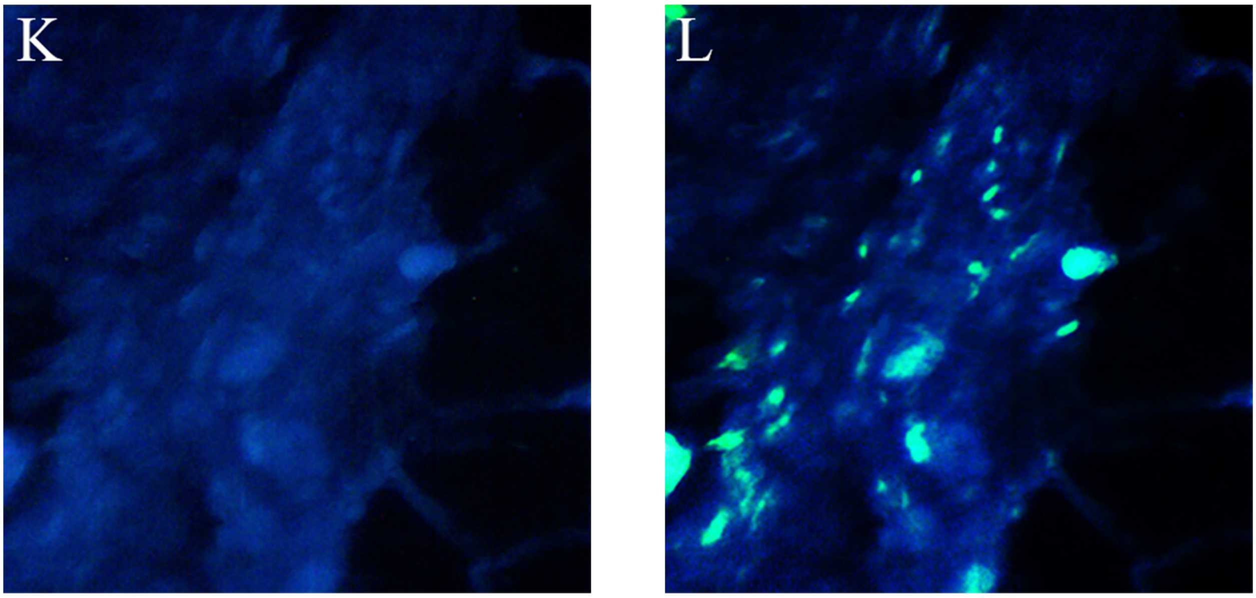

Supplementary Figure S5. MMP2 gene expression in Sham 1 (A, B, C), Sham 2 (D, E, F), Exp1 (G, H, I), andExp2 (J, K, L). A, D, G, J: primary antibody to mmp2; B, E, H, K: nuclei stained by DAPI; C, F, I, L: (Merging antibody and stained nuclei). Magnification: 200×.

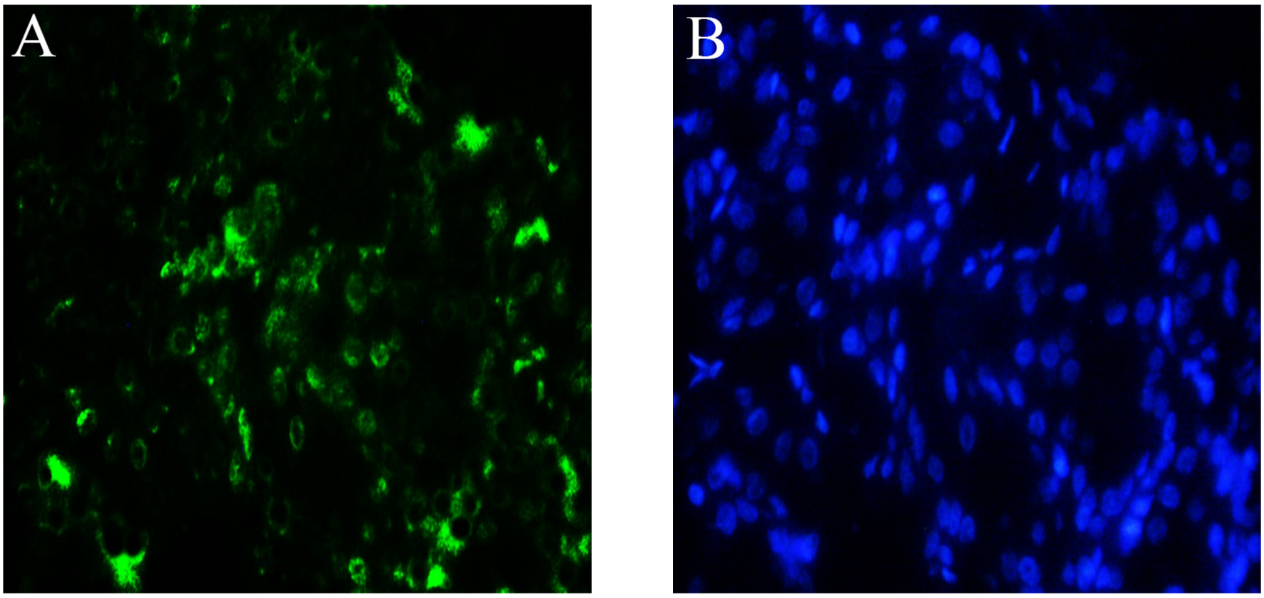

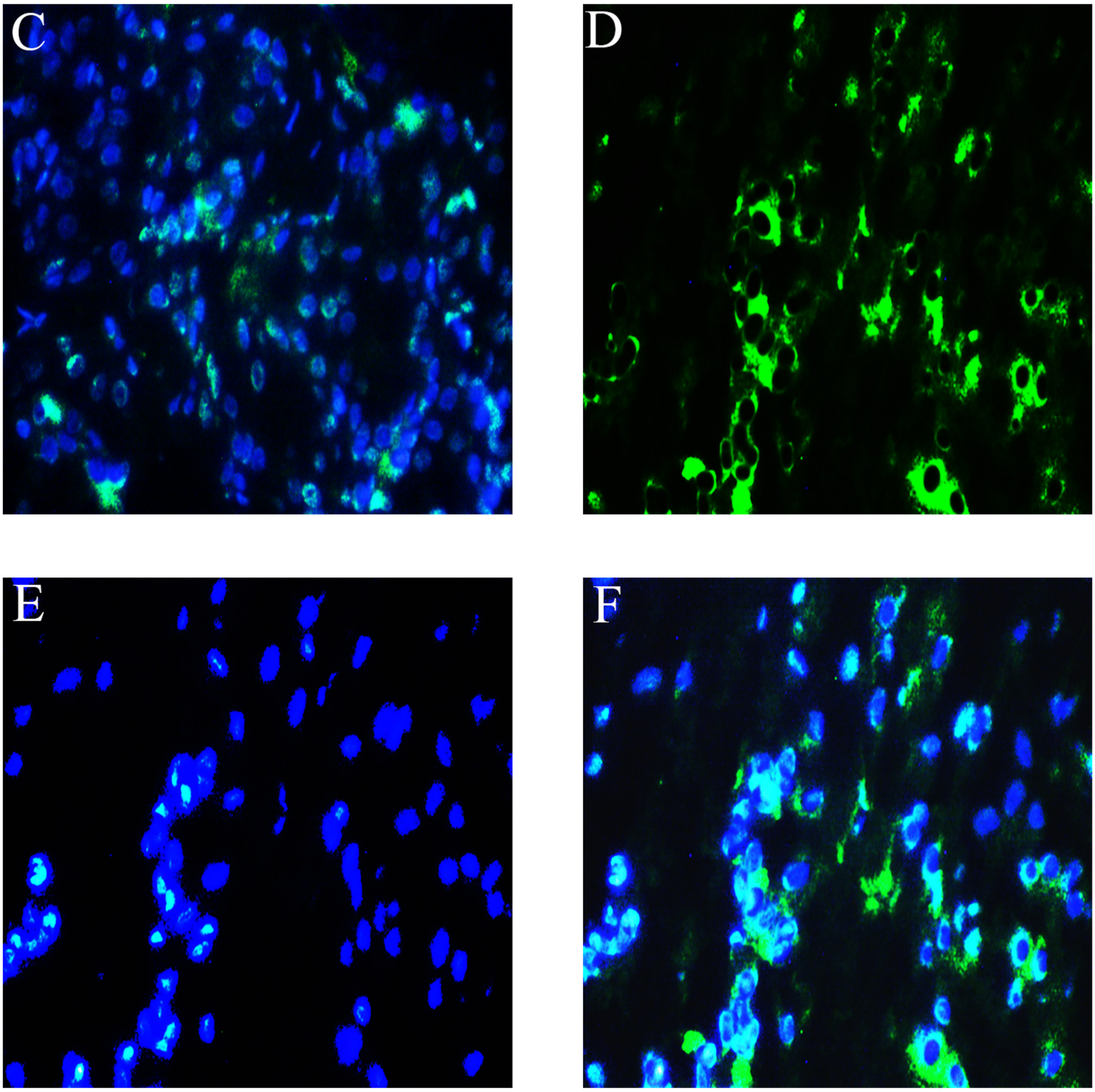
C
D
E
F

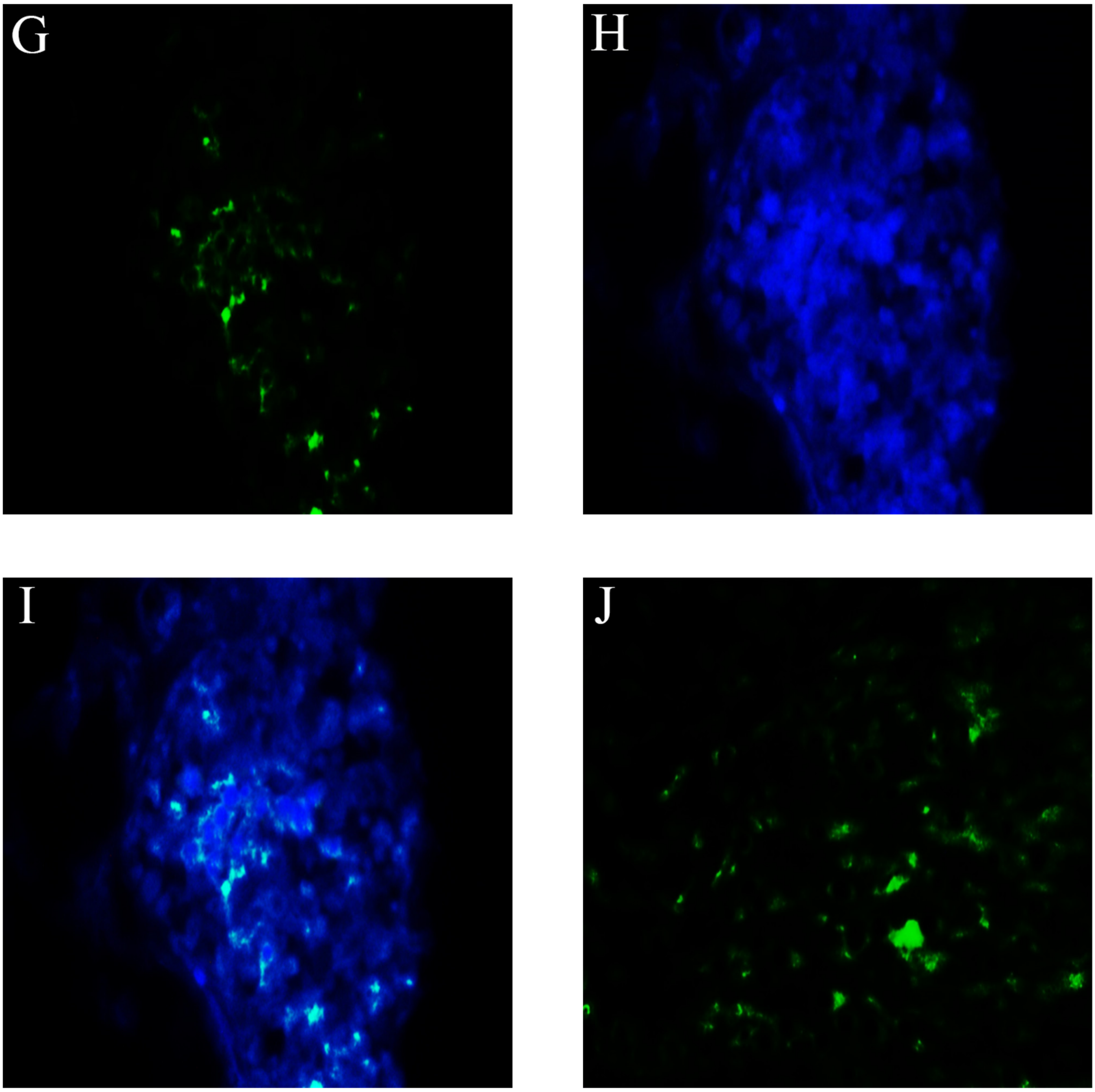
G
H
I
J

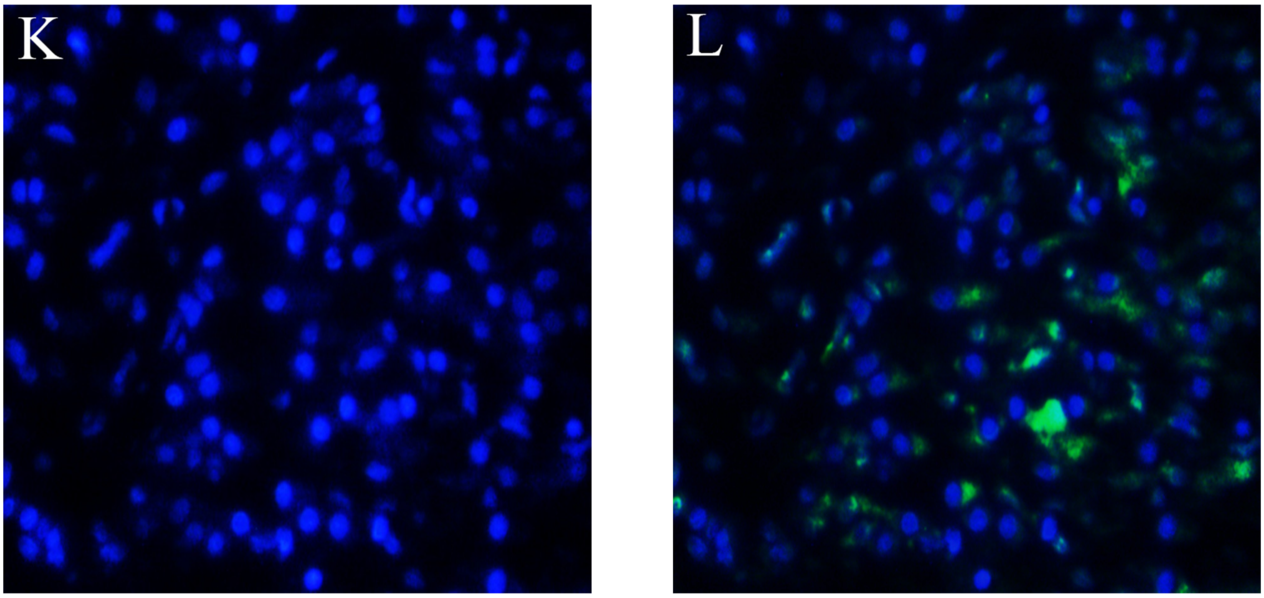

Supplementary Figure S6. TNF-α gene expression in Sham 1 (A, B, C), Sham 2 (D, E, F), Exp1 (G, H, I), andExp2 (J, K, L). A,D,G,J:primary antibody to mmp2;, B,E,H,K: nuclei stained by DAPI; C,F,I,L: (Merging antibody and stained nuclei).Magnification: 200×.

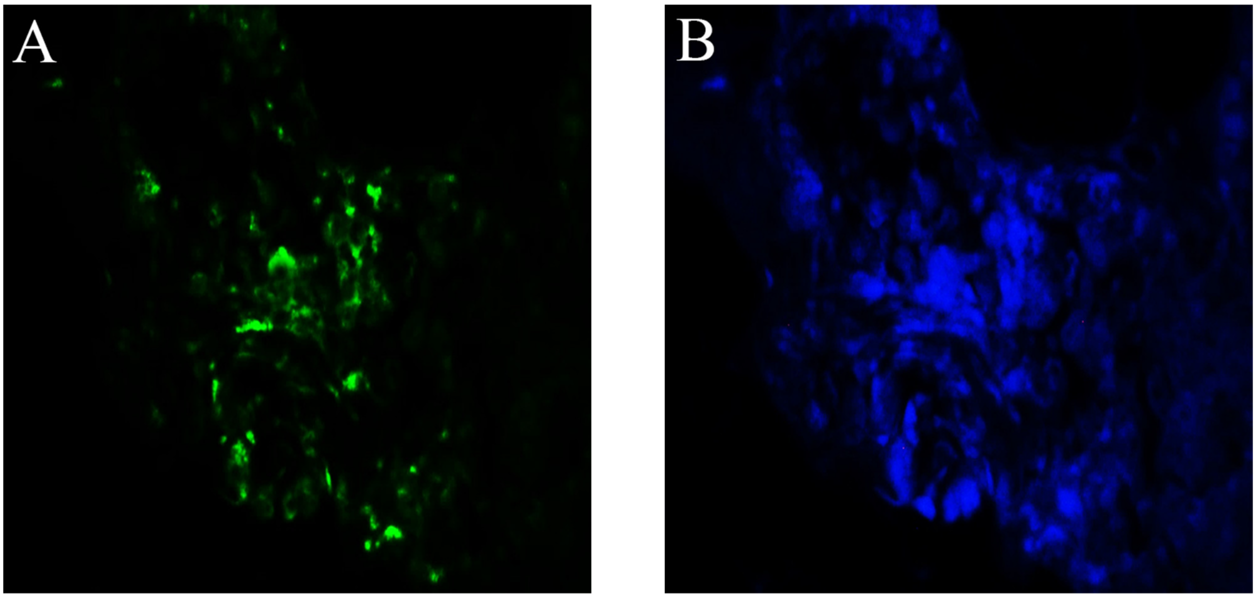

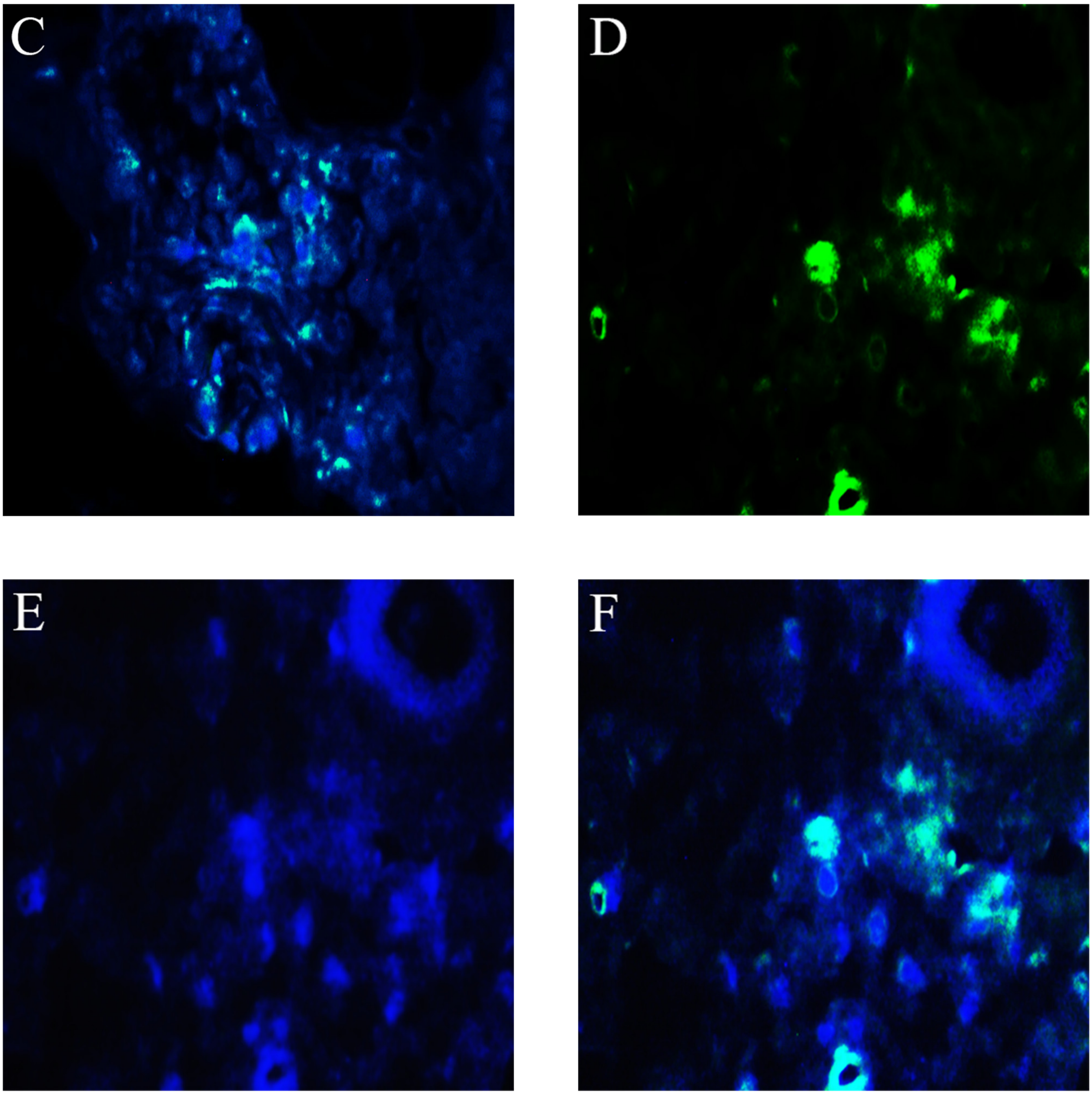
C
D
E
F

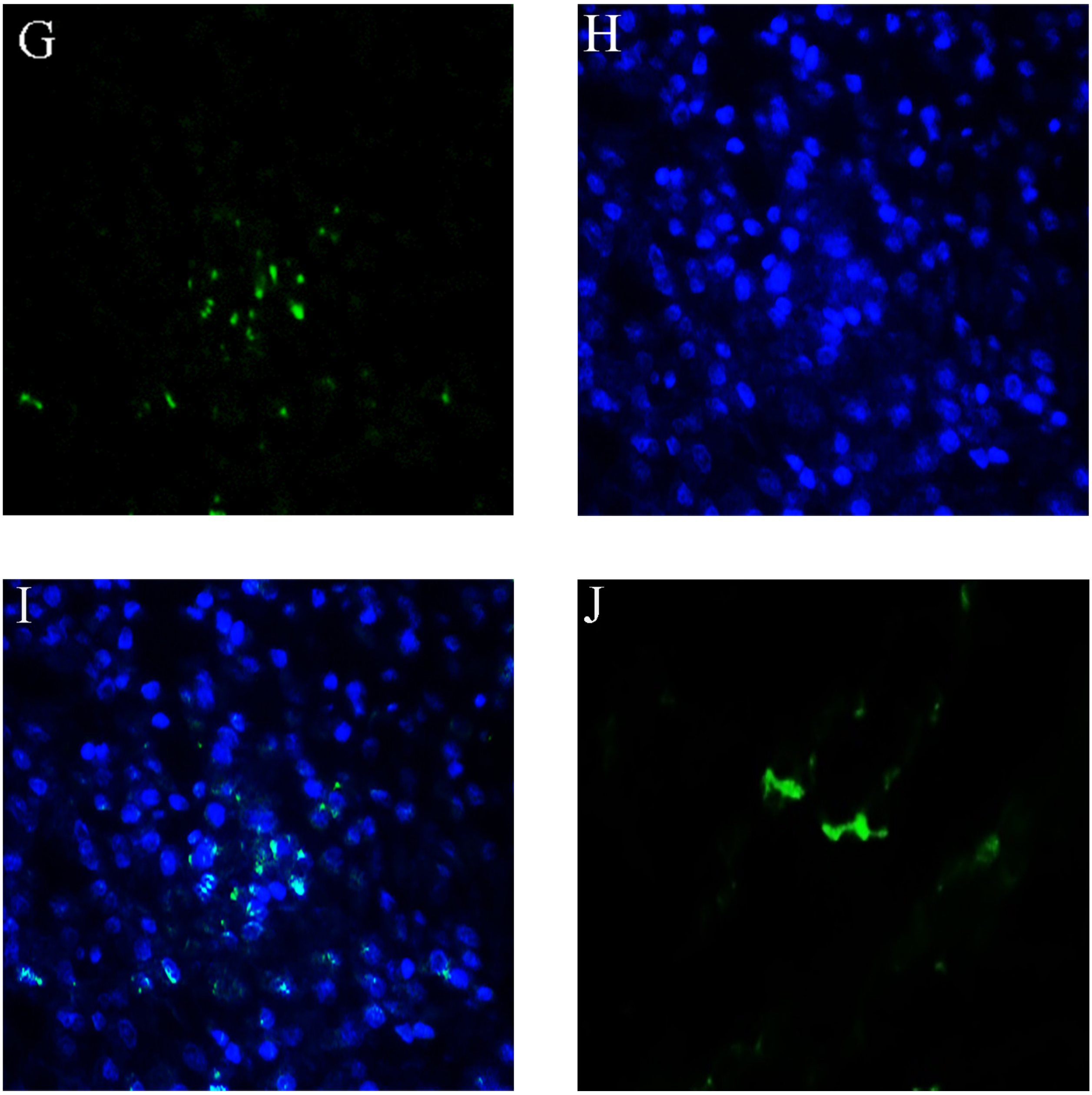
G
H
I
J

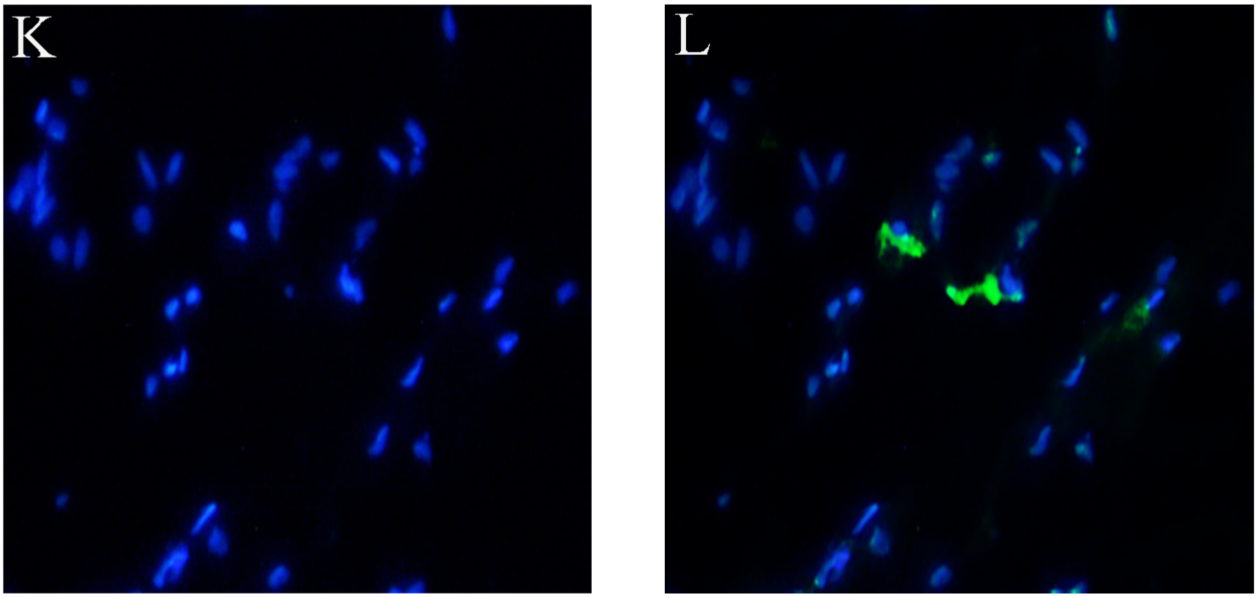

Supplementary Figure S7. IL-6 gene expression in Sham 1 (A, B, C), Sham 2 (D, E, F), Exp1 (G, H, I), andExp2 (J, K, L). A,D,G,J:primary antibody to mmp2; B,E,H,K: nuclei stained by DAPI; C,F,I,L: (Merging antibody and stained nuclei).Magnification: 200×.
